# Supplementary material for: Impact of higher-income countries on child health in lower-income countries from a climate change perspective. A case study of the UK and Malawi
Source: PLOS Glob Public Health. 2024 Jan 4;4(1):e0002721. doi: 10.1371/journal.pgph.0002721 (PMC10766172; doi:10.1371/journal.pgph.0002721)
Supplement: Attachment — Submitted filename: Response to Reviewers Climate change paper.docx [file pgph.0002721.s001.docx]

Dear Editor and Reviewers,

RE: PGPH-D-22-01397 ‘Impact of higher-income countries on child health in lower-income countries from a climate change perspective. Using the UK and Malawi as a case study’

Many thanks for taking the time to review our paper and give thorough and constructive feedback. We would like our paper to be considered for your ‘Climate Change and Human Health’ collection.

We have worked on the focus and organisation of the paper by putting more subheadings in and tables instead of prose. We believe this addresses the length that reviewer two felt was important. We do believe all original sections are relevant to give a thorough and complete picture of how a high-income country can impact a low-income country from a climate change perspective. We believe omitting any of these sections would give an incomplete picture.

In response to reviewer one’s comments:

1. We agree the structural roots of inequalities between and within countries lie in colonialism, imperialism and current global governance. We have explored these concepts in the introduction and incorporated subheadings to highlight this. We have put more focus on the global tax system which has its roots in colonialism and continues to create vast inequalities.
2. We have looked at the methods section and have tried to make it clearer. When starting this study, we felt the topic area was too broad to be a systematic review or scoping review and felt a critical appraisal of the literature was a more appropriate research design. Therefore, the review was conducted by the lead author. We have included the inclusion criteria, search engines used, grey literature search and dates of the search. We hope that is clearer but we have not gone into as much detail as would be expected for a scoping or systematic review.
3. Many thanks for your comments on how we can structure the results section. We have highlighted in the background section why figure one was used to structure the results section. We are aware of the length of our paper and repetition within the paper as highlighted by reviewer 2 and have chosen not to **begin the results with an overview of the literature collected and consequently used to derive the results**. We worry this would be repeating ourselves and adding length. We hope this is covered in the methods and reference section.
4. Many thanks for highlighting how important it is to emphasise the interconnection of how climate change will impact health in Malawi. We have added a paragraph at the end of this section drawing this together.
5. We agree making tables could make the adaptation section clearer and reduce the length of the paper as recommended by reviewer 2. To address this we have highlighted at the top of the table that these are projects Malawi receives climate finance for either indirectly through an international organisation that the UK government donates to or directly from part of the UK government eg Scotland’s climate justice fund. Where possible I have added dates and type of funding. We have also summarised the international organisations into a table.
6. A key and strong section of the results is the impact on UK climate policy on mitigation and children particularly the examination of the role of MNCs and the shortcomings in their report of carbon emissions. This section evidences the breadth of actors involved and their power and influences. **Many thanks for this comment we have highlighted the issue of the UK paying their fair share of adaptation finance and doing their fair share of climate mitigation. We believe this makes this section stronger.**
7. To elevate the discussion of the results further, it is recommended the results addressing each research question are synthesised further through the exploration of how the pathways indicated in the figure interact and influence one another. We have tried to do this throughout the discussion highlight that UK policies can influence the practices of MNCs and banks. We have also highlighted how MNCs and banks can influence government policies.
8. Additionally, the discussion section would benefit from an evaluation of the strengths and limitations of this research study. **Section added to discussion on this.**
9. The manuscript is clear and coherent however there are omissions in grammar in places, the table has no title/caption and there are inconsistencies in referencing style. We have addressed this.

**In response to Reviewer 2 Comments**

We have addressed the length of the paper by replacing some of the prose with tables, we have also taken out the international organisations and made a point of reducing the repetition. By incorporating the recommendations from reviewer one such as highlighting colonialism, imperialism and global governance we believe we have strengthened the argument that UK policies do impact child health in Malawi. We would like to summarise some key examples of why we feel there is a strong argument that UK policies impact child health in Malawi. If the UK does not do everything it can to mitigate against climate change we are contributing to the adverse health impacts in Malawi and can be held responsible for this for example with the UNCRC ruling. The UK does not contribute its fair share to climate finance which would help Malawi adapt to climate change. The UK facilitates a third of global tax abuse which deprives the Malawian government of vital revenue it can use to adapt to climate change. Individuals who have access to clean water, education and sanitation are less vulnerable to the impacts of climate change. Malawi loses about US$60 million each year to tax abuse, this amount could allow 17,500 children to access basic sanitation, 19,600 to access clean water and 541,000 children to spend an additional year in school. All of this are critical determinants for health. We have changed the title to make it more appropriate to the paper.

**Response to academic editor**

Many thanks for your comments. We have addressed the focus and organisation of the paper by making it much more UK Malawi focused. We have cut out some of the generic sections such as the part on international finance organisations and believe this makes the paper more readable.

Since the paper was initially submitted we have a new research assistant with expertise tax justice and imperialism. These were key recommendations to include from reviewer one. She has made a significant contribution to the paper and all authors are happy for her to be listed as second author. As the first author (EH) is commencing maternity leave we have made Rachel the corresponding author.

All authors of this paper have declared that no competing interests exist.

Funding for this work comes from NHS Education Scotland (EH) and The Professor Sonia Buist Global Child Health Research Fund (BOH). NHS Education Scotland pays the salary for Dr Eilish Hannah. The funders had no role in study design, data collection and analysis, decision to publish, or preparation of the manuscript.

Many thanks again for your feedback on our manuscript,

Kind Regards,

Dr Eilish Hannah
